# Partitioned airs at microscale and nanoscale: thermal diffusivity in ultrahigh porosity solids of nanocellulose

**DOI:** 10.1038/srep20434

**Published:** 2016-02-02

**Authors:** Koh Sakai, Yuri Kobayashi, Tsuguyuki Saito, Akira Isogai

**Affiliations:** 1 Department of Biomaterials Science, The University of Tokyo, Tokyo 113-8657, Japan

## Abstract

High porosity solids, such as plastic foams and aerogels, are thermally insulating. Their insulation performance strongly depends on their pore structure, which dictates the heat transfer process in the material. Understanding such a relationship is essential to realizing highly efficient thermal insulators. Herein, we compare the heat transfer properties of foams and aerogels that have very high porosities (97.3–99.7%) and an identical composition (nanocellulose). The foams feature rather closed, microscale pores formed with a thin film-like solid phase, whereas the aerogels feature nanoscale open pores formed with a nanofibrous network-like solid skeleton. Unlike the aerogel samples, the thermal diffusivity of the foam decreases considerably with a slight increase in the solid fraction. The results indicate that for suppressing the thermal diffusion of air within high porosity solids, creating microscale spaces with distinct partitions is more effective than directly blocking the free path of air molecules at the nanoscale.

High porosity solids are thermally insulating. Representative examples include plastic foams^1^ and silica aerogels^2^,^3^. Foams have microscale pores with a wall-like solid phase, whereas aerogels have nanoscale pores formed with a network-like skeleton of interconnected nanoparticles. The thermal conductivities *k* (W m^−1 ^K^−1^) of foams are slightly higher than that of atmospheric air^4^. Yet, their thermal diffusivities *α* (m^2 ^s^−1^) are one or two orders of magnitude lower. The relaxation of the temperature gradient within foams is thus a slow process; accordingly, foamed solids can function as heat insulators. In contrast, aerogels feature lower thermal conductivities than air^3^,^5^. This phenomenon is explained by the size of their pores, which are smaller than the mean free path of air molecules (~70 nm). The nanoscale network skeleton of aerogels inhibits the thermal diffusion of gases, so that the conductivity of the gas phase in aerogels becomes significantly lower when compared with that of atmospheric air.

Engineering of heat insulators is typically to design the pore structure of high porosity solids for controlling the heat energy transfer^5^,^6^,^7^,^8^,^9^. Therefore, it is important to determine the relationships between multiscale pore structures and their heat transfer properties. For a direct comparison of the heat transfer modes in various pores, it is essential to prepare high porosity solids with different pore sizes and shapes to be similar in solid fraction and chemical composition, as well as reducing the heat transport via solid phases as much as possible. However, a solid composition that can meet these requirements has not been reported until recently, and the heat transfer properties of foams and aerogels have been separately investigated^5^-^9^.

In the present study, we examined *α* and *k* of foams and aerogels with very low solid volume fraction, *V*_S_, values of 0.3-2.7%. These two porous structures are solely composed of about 3-nm-wide, crystalline cellulose nanofibers (CNFs). CNF is a skeletal component of trees, or representative porous structures in nature, and has recently been attracting attention as an excellent, bio-based nanomaterial with high strength, high stiffness, and low thermal expansion^9^-^12^. CNFs that are purified from wood can be individually dispersed in water via a specific surface-carboxylation reaction^13^,^14^. Both the foams and aerogels studied herein were prepared from aqueous dispersions of CNFs.

## Results

### Structures of the ultrahigh porosity solids of nanocellulose

**Figure 1:**
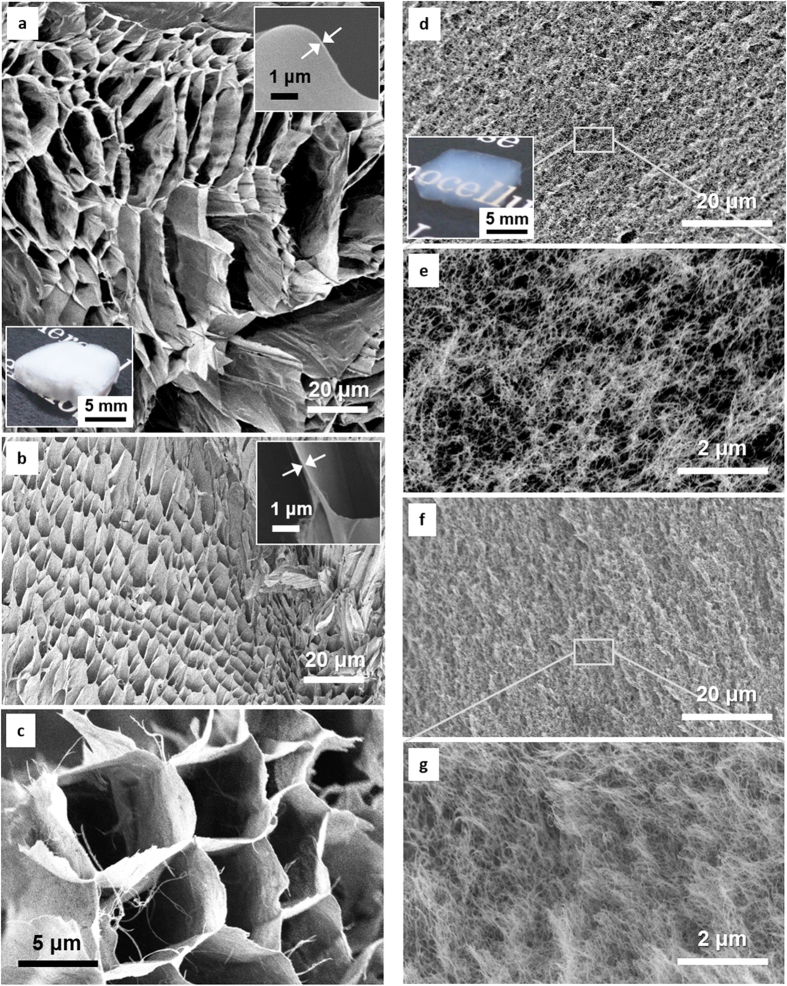
Scanning electron microscopy (SEM) images of the ultrahigh porosity foams and aerogels. (**a**) Foam with a solid volume fraction *V*_S_ of 0.32%. The upper right inset shows the thickness of the film-like solid phase at 0.32%, and the lower left inset shows the physical appearance of the foam. (**b**,**c**) Foam with a *V*_S_ of 1.04%. The inset in b shows the thickness of the film-like solid phase at 1.04%. (**d**,**e**) Aerogel-1 with a *V*_S_ of 0.60%. The inset in d shows the physical appearance of Aerogel-1. (**f**,**g**) Aerogel-1 with a *V*_S_ of 1.77%.

The foam samples were prepared by freeze-drying of the CNF/water dispersions^15^,^16^. When the dispersion is immersed in liquid nitrogen, microscale ice crystals grow from all around the dispersion, and CNFs are localized on the crystallite surfaces. Sublimation of the ice crystals results in the formation of a foamed structure with a film-like solid phase of the localized CNF aggregates (Fig. 1a–c, *V*_S_ 0.3-1.3%). The foam with the lowest *V*_S_ (~0.3%) had pores with heterogeneous sizes and shapes, and the cross-sectional sizes of the pores ranged from several μm to over 100 μm (Fig. 1a and Supplementary Fig. S1a). As the solid fraction increased, more pores with sizes of about 10 μm developed (Fig. 1b,c and Supplementary Fig. S1b). The thickness of the film-like CNF aggregates was roughly 100 nm, irrespective of the solid fraction (insets of Fig. 1a,b).

The aerogel samples were prepared via two different processes^17^,^18^. The first set of samples, denoted as Aerogel-1, was prepared via acid-induced gelation of the CNF dispersion, followed by solvent exchange from acidic water to ethanol, and supercritical CO_2_ drying^17^. Aerogel-1 samples displayed a nanoscale network-like skeleton of well-individualized CNFs (Fig. 1d–g, *V*_S_ 0.6-2.7%). The second set of samples, denoted as Aerogel-2, was prepared via *tert*-butanol-induced gelation of the dispersion, followed by solvent exchange from water/*tert*-butanol mixture to pure *tert*-butanol, and freeze drying^18^. The skeleton of Aerogel-2 was also network, but consisted of relatively aggregated CNFs (Supplementary Fig. S2, *V*_S_ 0.3-1.4%).

**Figure 2:**
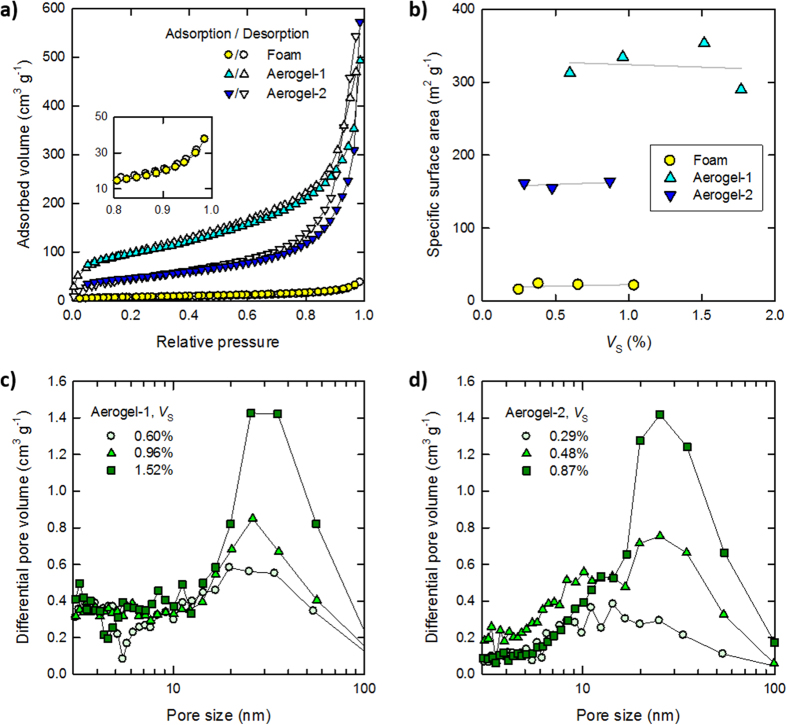
Nitrogen gas adsorption analyses. (**a**) The adsorption–desorption isotherms of the foam, Aerogel-1, and Aerogel-2 samples, with respective *V*_S_ values of 1.04%, 0.87%, and 1.52%. The inset shows an enlarged view of the plot of the foam. (**b**) Variations in the specific surface areas of the foam and aerogel samples as a function of *V*_S_. (**c**,**d**) Pore size distributions of the aerogels with different *V*_S_.

Figure 2a shows the nitrogen adsorption–desorption isotherms of the foam and aerogel samples. The amount of nitrogen gas adsorbed into the foam was low, and the isotherm showed no hysteresis between the adsorption and desorption processes (see inset). These results indicate that the solid phase of the foam, or the film-like CNF aggregate with a thickness of about 100 nm, have a dense and non-porous structure^19^,^20^. It has been reported that densely assembled CNF films show a very low gas permeability even at a thickness of 100 nm^21^. Thus, the film-like solid phase of the foam is expected to behave as a distinct barrier to the thermal diffusion of gases. Contrarily, both types of aerogels adsorbed large amounts of nitrogen gas, and their isotherms showed a distinct hysteresis at the high relative pressures. This hysteresis indicates the presence of nanoscale pores^19^,^20^, as consistent with the microscopy analysis (Fig. 1d–g).

Figure 2b shows the specific surface areas of the samples estimated from the isotherms. The specific surface areas of the foams, Aerogel-1, and Aerogel-2 were independent of *V*_S_, and were approximately 20, 300-350, and 160 m^2^ g^−1^, respectively. In general, solids with large specific surface areas show lower thermal conductivities^22^. Thus, the thermal conductivities of the solid phases are assumed to be in the order of Aerogel-1< Aerogel-2< foam at a given solid fraction, and linearly increase as the fraction of each solid phase increases^5^,^6^. Figure 2c,d show the pore size distributions of the Aerogel-1 and Aerogel-2 samples, respectively, as estimated from the isotherms. The pore sizes of both types of aerogels ranged from a few nm to over 100 nm, and more pores with sizes of about 30 nm developed as the solid fraction increased.

### Comparison of the heat transfer properties

**Figure 3:**
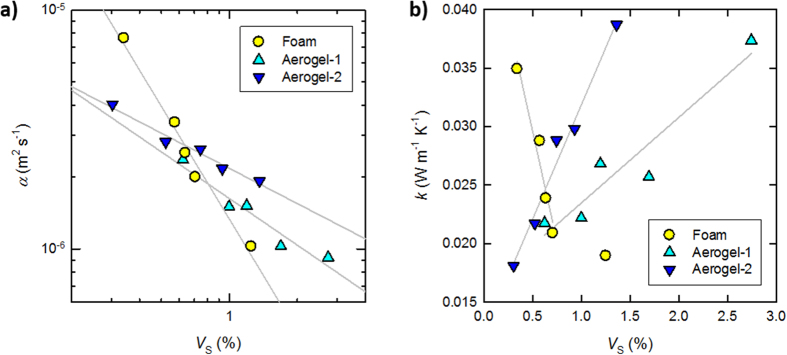
The heat transfer properties of the foam and aerogels. (**a**) Variations in the diffusivities *α* of the form and aerogels as a function of *V*_S_. (**b**) Variations in the conductivities *k* of the form and aerogels as a function of *V*_S_.

Figure 3a shows the thermal diffusivities *α* of the foam and aerogels as a function of *V*_S_. It is noteworthy that both the foam and aerogel samples showed linear relationships in the log–log plot. These empirical relationships can be described as follows,


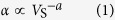


where exponent *a* corresponds to the slope of the log–log plot, and shows the dependency of thermal diffusivity *α* on *V*_S_. The exponents *a* of the foam, Aerogel-1, and Aerogel-2 were 1.6, 0.6, and 0.5, respectively. Compared with the thermal diffusivity of aerogels with *a* = 0.5-0.6, the thermal diffusivity of the foam reduced considerably with increasing *V*_S_. In fact, the solid phase of the foam contributes more effectively to the delay of the temperature gradient relaxation when compared with the aerogels, and the exponent *a* reflects their pore structures (see also Supplementary Fig. S3).

Figure 3b shows the thermal conductivities *k* of the foam and aerogels, as calculated from their respective diffusivities *α* using the following equation,


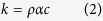


where *ρ* and *c* are the density (g m^−3^) and specific heat capacity (J g^−1 ^K^−1^) of a given sample, respectively. As observed, within a narrow *V*_S_ range of 0.3-0.7%, *k* of the foam decreased considerably from 0.035 W m^−1 ^K^−1^ to 0.021 W m^−1 ^K^−1^ (with the latter value being lower than that of atmospheric air (0.026 W m^−1 ^K^−1^)) despite increasing *V*_S_ of the highly conductive solid phase. This result was attributed to the strong dependency of the diffusivity on *V*_S_ (exponent *a* = 1.6). The air within a microscale pore transports heat by natural convection, so that the gas phase in the foamed structures has the same conductivity as atmospheric air (Supplementary Fig. S3). However, it is assumed that heat transfer between the gas and solid phases significantly contributes to the reduction of the total conductivity *k* for high porosity foams with microscale closed pores^23^. This contribution is neglected for common insulating foams, pores of which are coherent^6^; accordingly, their *k* values are inevitably higher than that of atmospheric air. The heat transfer between the gas and solid phases is described based on Newton’s law of cooling as follows^24^,


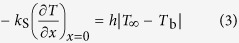


where *k*_S_ is the thermal conductivity of the solid phase, (∂*T*/∂*x*)_x = 0_ is the temperature gradient at the boundary surface, *h* is the heat transfer coefficient (W m^−2 ^K^−1^), the inverse of which is the interfacial thermal resistance, *T*_∞_ is the equilibrium temperature of the gas phase, and *T*_b_ is the temperature at the boundary surface. In equation (3), the left and right members describe the local heat fluxes of the solid and gas phases, respectively, at the boundary surface. Although *k*_S_ is specific to the solid, or the film-like CNF aggregate (0.11 W m^−1 ^K^−1^), *h* is variable and strongly depends on the flow of the gas phase^24^. Thus, the gas phase in a microscale closed pore should show a low *h* and a significant temperature difference |*T*_∞_ − *T*_b_|, which is supported by the fact that the relaxation of the temperature gradient within the foam was markedly delayed by the film-like solid phase (Fig. 3a). As the pore sizes of the foam decrease with increasing *V*_S_ (Fig. 1a,b), the contribution of the interfacial heat transfer to *k* become large^23^, thus explaining the significant reduction of *k* to that below that of air. It has also been reported elsewhere that foamed CNF structures show very low thermal conductivities^9^,^25^.

In contrast, the thermal conductivities of both types of aerogels increased with increasing *V*_S_. This is a general phenomenon that can be directly explained where the heat transfer between the gas and solid phases is negligible^5^; that is, the thermal conductivities of the solid and gas phases of aerogels with nanoscale open pores can be separately estimated. Accordingly, *k* of the aerogels increased with increasing *V*_S_. It should also be noted that *k* of Aerogel-1 increased more steadily when compared with the increase observed for Aerogel-2. This is most likely to arise from the larger specific surface areas of the Aerogel-1 solid skeleton (see Fig. 2b and related descriptions).

The radiative heat transfer is not taken into account in the comparison studies of the foam and aerogels^5^,^6^. The amplitude of the thermal waves applied to the samples in the thermal diffusivity measurements was very small (approximately ±0.5 °C at 23 °C), and all the porous solids prepared in the present study featured the same chemical composition, a thin sample thickness (~3 mm), and a very low solid fraction. Hence, the contribution of radiative heat transfer is negligible.

## Discussion

We have directly compared the heat transfer properties of very high porosity foams and aerogels. The foams featured rather closed, microscale pores with a thin film-like solid phase of CNF aggregates, whereas the aerogels featured nanoscale open pores with a network-like solid skeleton of CNFs. The thermal diffusivity *α* of the foam markedly decreased with slight increases in the solid fraction when compared with those observed in the aerogels. The decrease in *α* of the foam is explained by the interfacial thermal resistance *h*^−1^ of air against the film-like solid phase^23^, whereas that for the aerogel is based on the blocking of the free path of air molecules (~70 nm) by the nanofibrous solid skeleton (see Supplementary Fig. S3)^5^. In fact, the present results indicate that for suppressing the thermal diffusion of air or a gaseous fluid within highly porous structures, creating microscale spaces with distinct partitions is more effective than directly blocking collisions between air molecules at the nanoscale. These findings may contribute to the understanding or control of diffusion-limited, various microfluidic systems including not only heat insulators but also microreactors and plant tissues, thereby enabling more efficient processes.

## Methods

### Preparation of CNF/water dispersions

CNF dispersions were prepared from a softwood bleached kraft pulp according to a previously reported protocol^13^. Briefly, the pulp (1 g) was chemically modified via a specific surface-carboxylation reaction in water (100 mL) under ambient conditions using 2,2,6,6-tetramethylpiperidine-1-oxyl (TEMPO) as the catalyst (1 mmol L^−1^) combined with sodium bromide (10 mmol L^−1^) and sodium hypochlorite (10 mmol g^−1^ of the pulp). Then, the modified pulp (carboxylate content: 1.7 mmol g^−1^) was suspended in water at a concentration of 0.5% w/v, and disintegrated into CNFs by passing the suspension through a high-pressure water jet system (HJP-25001, Sugino Machine) five times. The concentrations of the resulting CNF dispersions were adjusted to 0.4–2% w/v by diluting with water or condensing with an evaporator at 45 °C under reduced pressure.

### Preparation of the foam samples

The foam samples were prepared from the 0.4-2% w/v CNF dispersions according to a previously reported method with a slight modification^16^. An aluminum container with inner dimensions of 10 mm × 5 mm × 3 mm was filled with the CNF dispersion, and immersed in liquid nitrogen at −196 °C. Then, the frozen sample was quickly subjected to vacuum drying at 2 Pa and ambient temperatures for 3 days to sublimate the ice within the frozen sample. The resulting foam samples had bulk densities of about 4-20 mg cm^−3^ at 23 °C and 50% relative humidity.

### Preparation of the Aerogel-1 samples

The Aerogel-1 samples were prepared from the 0.4–2% w/v CNF dispersions according to a previously reported method^17^. The CNF dispersion (10 mL) was poured into a plastic mold with inner dimensions of 6 cm × 5.5 cm × 1 cm. A 1 M HCl solution (10 mL) was spread over the dispersion and allowed to stand for 1 h. The resulting free-standing hydrogel was taken from the mold and shaken in a mixed solution of 0.01 M HCl (75 mL) and ethanol (75 mL) for 1 day. The hydrogel was then cut into pieces of about 10 mm × 5 mm × 3 mm in size using a sharp blade, and shaken in ethanol (150 mL) for 3 days with replacement of the solvent with fresh ethanol twice a day. The resulting alcogels were placed in the chamber of a critical point dryer (SYGLCP-8, Sanyu-Gijutsu) under a liquid CO_2_ flow (1 L min^−1^ by gasified volume) at 15 °C for 8 h. The chamber temperature was increased to 40 °C to obtain the supercritical phase, maintained for 30 min, after which the chamber was gradually depressurized for 1 h. The resulting Aerogel-1 samples had bulk densities of about 10-40 mg cm^−3^ at 23 °C and 50% relative humidity.

### Preparation of the Aerogel-2 samples

The Aerogel-2 samples were prepared from the 0.4–1% w/v CNF dispersions according to a previously reported method with a slight modification^18^. The CNF dispersion (10 mL) was well mixed with *tert*-butyl alcohol (20 mL), and centrifuged at 1,400 *g* for 10 min at about 30 °C. The resulting viscous precipitate (~8 mL) was again mixed with *tert*-butyl alcohol (30 mL) and centrifuged under identical conditions as above. This mixing–centrifugation treatment was repeated three times. The final precipitate was poured into an aluminum container with inner dimensions of 10 mm × 5 mm × 3 mm, and freeze-dried in the same way as that performed for the foam samples. The resulting Aerogel-2 samples had bulk densities of about 4–20 mg cm^−3^ at 23 °C and 50% relative humidity.

### Volume fraction of the solid phase

The solid volume fractions *V*_S_ (%) values of the samples were calculated from the bulk density *ρ* (g cm^−3^) values using the following equation,


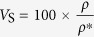


where ρ* is the true density of the CNF prepared in the present study (1.64 g cm^−3^) determined by helium pycnometry using a true density analyzer (BELPycno, Microtrac BEL). Although the samples contained moisture of about 5% w/w at 23 °C and 50% relative humidity, it was neglected in the calculation of *V*_S_.

### SEM analysis

The samples were sectioned using tweezers, and the resulting cross-sections of the samples were treated using a Meiwafosis Neo osmium coater at 5 mA for 10 s. The thickness of the osmium coating layers was estimated as 2.5 nm thick under the conditions employed. These samples were then examined by SEM on a Hitachi S-4800 field-emission microscope, operating at 1 kV.

### Nitrogen adsorption-desorption measurement

The nitrogen adsorption**-**desorption isotherms of the samples were measured on a Quantachrome NOVA 4200e at −196 °C after degassing of the samples in the system at 105 °C for 20 h. The specific surface areas and pore size distributions of the samples were estimated from the isotherms using the Brunauer–Emmett–Teller (BET) theory and the Barrett-Joyner-Halenda (BJH) model, respectively^19^,^20^.

### Thermal diffusivity measurement

The thermal diffusivities of the samples were measured at 23 °C and 50% relative humidity by temperature wave analysis using an ai-Phase Mobile 1u device equiped with a sensor of 0.5 mm × 0.2 mm in size. The sample, with a thickness of about 3 mm, was sandwiched between the heater and sensor plates, and the distance between the two plates was adjusted to create a firm contact with both surfaces of the sample. The adjusted distance, or thickness of the slightly compressed sample, was monitored within an accuracy of ±1 μm, and the sample density *ρ* was thus corrected using the distance. The applied voltage was set to 1.4 V, and the delay in phase of the temperature wave (amplitude of about ±0.5 °C) was measured at eight points within a range of 0.2–2 Hz. The integration time for each point was typically 70 s. During the measurement, the entire device was covered with an optically opaque windshield. The contribution of the boundary layers between the sample and the heater/sensor plate to the diffusivities α is negligible, because the samples were compressed by about 1–5% between the heater and sensor plates, and the boundary layers are assumed to be thin enough (of order 1 μm or less) as compared to the sample thickness ~3 mm. The specific heat capacity *c* at around 23 °C, which was used for calculating *k*, was 0.90 J g^−1 ^K^−1^, as determined by differential scanning calorimetry using a Perkin-Elmer DSC 8500 instrument (Supplementary Fig. S4). For the measurement, the sample was conditioned at 23 °C and 50% relative humidity and packed into an aluminum pan.

## Additional Information

**How to cite this article**: Sakai, K. *et al*. Partitioned airs at microscale and nanoscale: thermal diffusivity in ultrahigh porosity solids of nanocellulose. *Sci. Rep.* **6**, 20434; doi: 10.1038/srep20434 (2016).

## Supplementary Material

Supplementary Information
