# Supplementary material for: Partitioned airs at microscale and nanoscale: thermal diffusivity in ultrahigh porosity solids of nanocellulose
Source: Sci Rep. 2016 Feb 2;6:20434. doi: 10.1038/srep20434 (PMC4735846; doi:10.1038/srep20434)
Supplement: Supplementary Information [file srep20434-s1.pdf]

Supplementary Information

Partitioned airs at microscale and nanoscale: thermal diffusivity in ultrahigh porosity solids of nanocellulose

Koh Sakai, Yuri Kobayashi, Tsuguyuki Saito* and Akira Isogai

Department of Biomaterials Science, The University of Tokyo, Tokyo 113-8657, Japan

*Corresponding author. E-mail: asaitot@mail.ecc.u-tokyo.ac.jp

This file includes Supplementary Figures S1−S4.

Figure S1: Low-magnification SEM images of the same foam samples as those in Figure 1a–c, showing the overall structures of their pores.

Figure S2: SEM images of the Aerogel-2 samples, showing their network-like skeletons consisting of relatively aggregated CNFs as compared to those of the Aerogel-1 samples.

Figure S3: Single logarithmic plots of the thermal diffusivities α, suggesting that the gas phase in the foam transports heat by natural convection, whereas that of the aerogels transports heat in a substantially different way.

Figure S4: Specific heat capacity c of the CNF, which was used on the calculation of the thermal conductivities k.

Experimental details are described in Methods section in the Article.

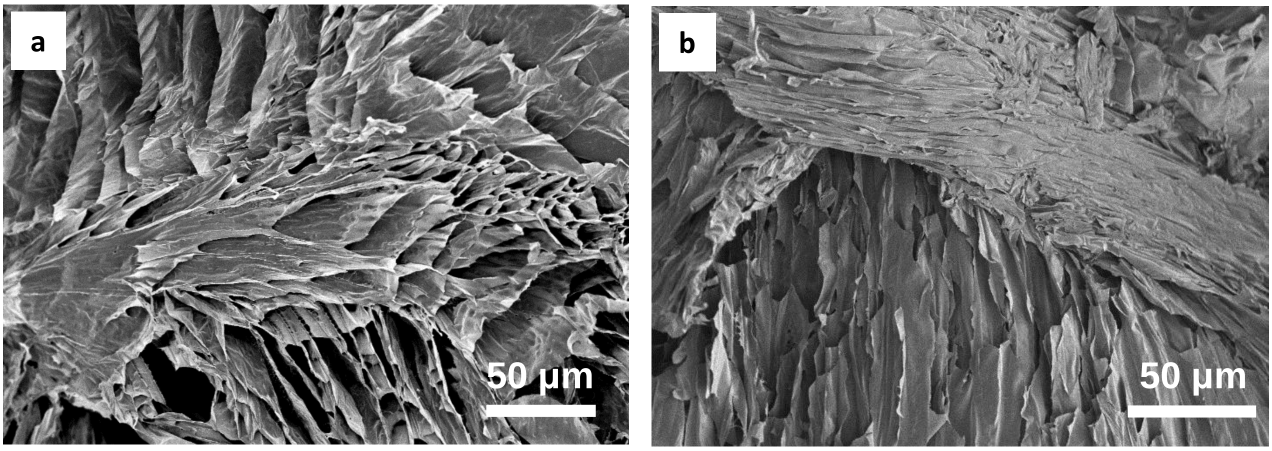

Figure S1. Low-magnification SEM images of the foam samples. (**a**) Foam with a V_S of 0.32%. (**b**) Foam with a V_S of 1.04%.

The foam samples were prepared by freeze-drying of the CNF/water dispersions. When the dispersion is immersed in liquid nitrogen, microscale ice crystals grow from all around the dispersion, and CNFs are localized on the crystallite surfaces (ref. 15). Figure S1 shows that the microscale ice crystals were columnar or layered, and locally oriented, but isotropic as a whole.

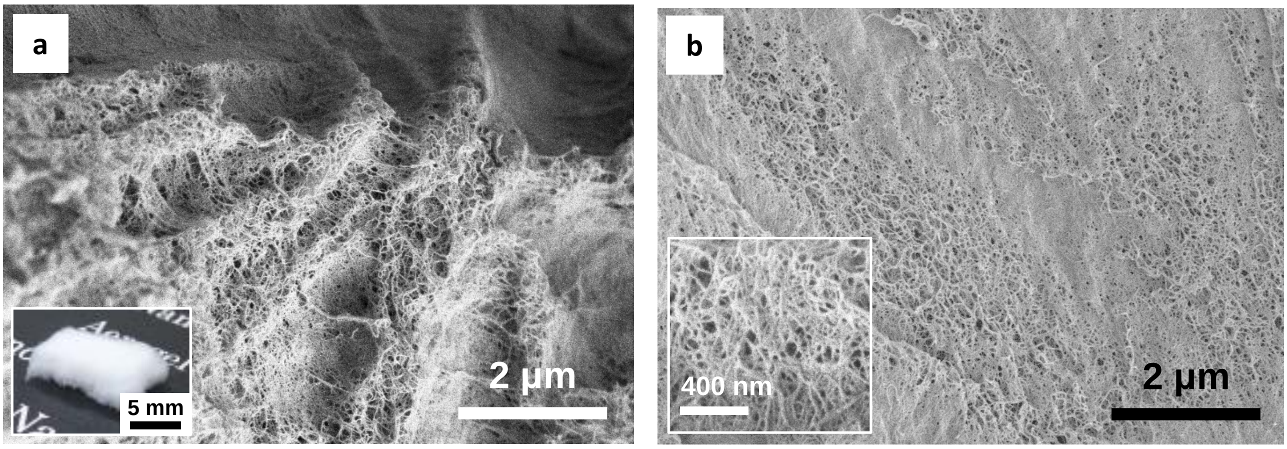

Figure S2. SEM images of the Aerogel-2 samples. (**a**) Aerogel-2 with a V_S of 0.48%. The inset shows the physical appearance of Aerogel-2. (**b**) Aerogel-2 with a V_S of 0.87%. The inset shows a high-magnification image of the cross section.

Figure S2 shows the skeletal structures of the Aerogel-2 samples. Their skeletons are nanoscale networks as similar to those of the Aerogel-1, but consisted of relatively aggregated CNFs as compared to those of the Aerogel-1 samples. The differences in specific surface area between the Aerogel-1 and Aerogel-2 samples are shown in Figure 2b.

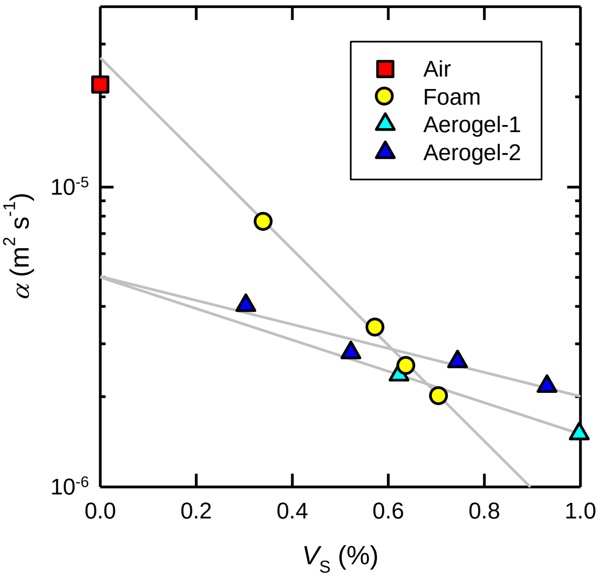

Figure S3. Single logarithmic plots of the thermal diffusivities α within a V_S range of 0−1%.

The approximated line (gray line) for the α values of the foams with V_S of 0.3−0.7% is extrapolated at V_S = 0 to an α value close to that of atmospheric air (red square), but this is not the case with the aerogels. These results indicate that the gas phase in the foam transports heat by natural convection, whereas that of the aerogels, showing a considerably lower extrapolated value than that of atmospheric air, transports heat in a substantially different way. The thermal diffusion of gas molecules within the aerogels is inhibited by the nanoscale network skeletons of the aerogels (ref. 5).

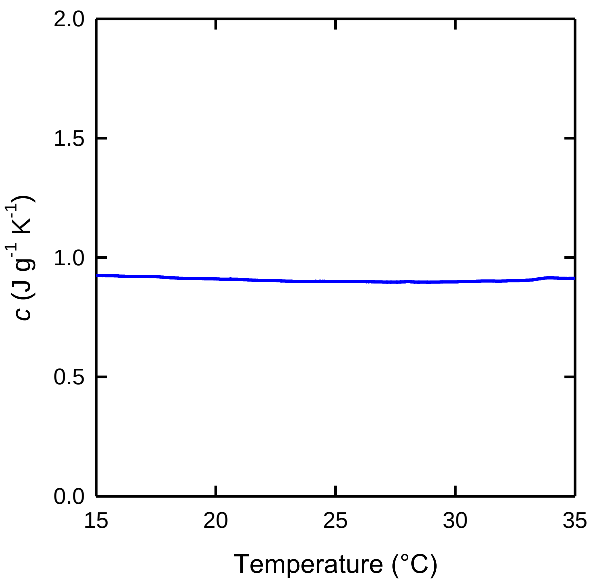

Figure S4. Specific heat capacity *c* of the CNF within a temperature range of 15−35 °C.

The thermal diffusivities of the samples were measured at 23°C. The specific heat capacity *c* at around 23°C was almost constant (0.90 J g^{-1} K^{-1}), showing no phase transition during the diffusivity measurements. The *c* value of 0.90 J g^{-1} K^{-1} was used for calculating *k* from the diffusivities α.
